# Transcriptomic Responses Underlying the High Virulence of Black Queen Cell Virus and Sacbrood Virus following a Change in Their Mode of Transmission in Honey Bees (*Apis mellifera*)

**DOI:** 10.3390/v15061284

**Published:** 2023-05-30

**Authors:** Yahya Al Naggar, Hassan Shafiey, Robert J. Paxton

**Affiliations:** 1 1 General Zoology, Institute for Biology, Martin Luther University Halle-Wittenberg, Hoher Weg 8, 06120 Halle (Saale), Germany; h.shafiey@gmail.com (H.S.); robert.paxton@zoologie.uni-halle.de (R.J.P.); 2 Zoology Department, Faculty of Science, Tanta University, Tanta 31527, Egypt; 3 Department of Community Ecology, UFZ-Helmholtz Centre for Environmental Research, Theodor-Lieser-Str. 4, 06120 Halle (Saale), Germany

**Keywords:** *Apis mellifera*, BQCV, SBV, virulence, innate immunity, RNAi, antiviral response, transcriptome

## Abstract

Background: Over the last two decades, honey bees (*Apis mellifera*) have suffered high rates of colony losses that have been attributed to a variety of factors, chief among which are viral pathogens, such as deformed wing virus (DWV), whose virulence has increased because of vector-based transmission by the invasive, ectoparasitic varroa mite (*Varroa destructor*). A shift in the experimental mode of transmission of the black queen cell virus (BQCV) and sacbrood virus (SBV) from fecal/food–oral (direct horizontal) to vector-mediated (indirect horizontal) transmission also results in high virulence and viral titers in pupal and adult honey bees. Agricultural pesticides represent another factor that acts independently or in interaction with pathogens, and they are also thought to cause colony loss. Understanding the molecular mechanisms underlying the higher virulence following a vector-based mode of transmission provides deeper insight into honey bee colony losses, as does determining whether or not host–pathogen interactions are modulated by exposure to pesticides. Methods: Through an experimental design with controlled laboratory, we investigated the effects of the modes of transmission of BQCV and SBV (feeding vs. vector-mediated via injection) alone or in combination with chronic exposure to sublethal and field-realistic concentrations of flupyradifurone (FPF), a novel agricultural insecticide, on honey bee survival and transcription responses by using high-throughput RNA sequencing (RNA-seq) analysis. Results: Co-exposure to viruses via feeding (VF) or injection (VI) and FPF insecticide had no statistically significant interactive effect on their survival compared to, respectively, VF or VI treatments alone. Transcriptomic analysis revealed a distinct difference in the gene expression profiles of bees inoculated with viruses via injection (VI) and exposed to FPF insecticide (VI+FPF). The number of differentially expressed genes (DEGs) at log2 (fold-change) > 2.0 in VI bees (136 genes) or/and VI+FPF insecticide (282 genes) was very high compared to that of VF bees (8 genes) or the VF+FPF insecticide treatment (15 genes). Of these DEGs, the expression in VI and VI+FPF bees of some immune-related genes, such as those for antimicrobial peptides, Ago2, and Dicer, was induced. In short, several genes encoding odorant binding proteins, chemosensory proteins, odor receptors, honey bee venom peptides, and vitellogenin were downregulated in VI and VI+FPF bees. Conclusions: Given the importance of these suppressed genes in honey bees’ innate immunity, eicosanoid biosynthesis, and olfactory associative function, their inhibition because of the change in the mode of infection with BQCV and SBV to vector-mediated transmission (injection into haemocoel) could explain the high virulence observed in these viruses when they were experimentally injected into hosts. These changes may help explain why other viruses, such as DWV, represent such a threat to colony survival when transmitted by varroa mites.

## 1. Introduction

Honey bees (*Apis mellifera*) play an important role in crop and wild plant pollination [1,2,3]. However, they have suffered overwinter colony losses since 2006, particularly in Europe and the United States [4,5,6]. A variety of biotic and abiotic factors, such as parasites, pathogens, inadequate nutrition, and pesticide exposure, can act alone or in combination to cause honey bee colony losses [7,8,9]. Among the pathogens are viruses, some of which are spread by exotic ectoparasitic varroa (*Varroa destructor*) mites, which are generally known as a major cause of colony death and can play a role with other biotic and abiotic stressors to cause the collapse of host colonies [10].

Viruses can spread horizontally, vertically, or by both modes of transmission [11]. However, the arrival of the varroa vector in honey bee populations has caused a shift in the transmission of some viruses that are now transmitted indirectly through an intermediate biological vector rather than directly through food or contact with other infected hosts. For example, the global spread of varroa has resulted in the emergence of DWV variants, allowing it to become one of the most widespread insect viruses on the planet [12,13]. The tracheal mite, *Acarapis woodi*, may also play a role as an additional potential vector for picorna-like viruses [14], though this is unknown. This change in the mode of transmission is expected to result in a change in parasite virulence (the damage incurred by the host as a result of infection), though the direction of the change is not always easy to predict [15]. Because vector-based transmission changes the evolutionary trade-off between virulence and transmission, it has the potential to increase the virulence [16]. In addition, vector-based transmission can also directly result in an increase in virulence because viral particles are now directly injected into a host rather than having to pass through defensive barriers, such as the digestive system [17]. An increase in virulence following a change in the transmission route was documented in an isopod and its endosymbiont *Wolbachia* [18], as well as in honey bees and their viruses: the black queen cell virus (BQCV) and sacbrood virus (SBV) [19,20].

BQCV represents one of the most prevalent honey bee viruses [21,22,23,24]. It is horizontally transmitted by nurse honey bees from infected to healthy larvae in brood food [25], and queens may also vertically transmit it to eggs [11]. SBV is yet another virus that infects both Western and Eastern honey bees (*Apis mellifera* and *Apis cerana*). It can infect both the brood and adult stages of the honey bee, but it is most pathogenic to two-day-old larvae [26]. SBV transmission in honey bee colonies can occur through either vertical or horizontal transmission routes [10,27]. It was also found to be transmitted venereally in honey bees, where the virus was passed on to queens via drone sperm during mating or artificial insemination [28,29]. BQCV and SBV may commonly co-occur in honey bees [20].

Although both BQCV and SBV are not known to be transmitted by *V. destructor*, many studies have found a significantly higher prevalence of BQCV [24,30] and SBV [30,31,32,33] in colonies infested with *V. destructor* mites than in those that are free of mite infestation, suggesting a potential role—direct or indirect—of *V. destructor* in the transmission of both BQCV and SBV. Though there has been no evidence of BQCV and SBV replication in *V. destructor*, implying that the mites may merely act as mechanical carriers of the virus [30,32], the shift in the mode of experimental transmission of BQCV and SBV from fecal/food–oral (direct horizontal) to vector-mediated (indirect horizontal) transmission has been shown to result in a significant increase in mortality and the viral titer of pupae [20] and adult honey bees [19,34]. A change to vector-borne transmission of BQCV and SBV therefore poses a future potential threat to bees and apiculture. Understanding the molecular mechanisms underlying the high virulence of these viruses following experimental changes in their mode of transmission is important not only for exploring future threats, but also for informing on the biological impacts of viruses on honey bee hosts.

Pesticides have been shown to promote pathogen replication in hosts [35]. For example, honey bees exposed to the novel insecticide flupyradifuorane (FPF) and the bee pathogen *N. ceranae* died more rapidly and experienced altered expression of immune and detoxifying genes [36]. The insecticide FPF has a mode of action similar to that of neonicotinoids, which are selective agonists of nicotinic acetyl choline receptors (NAChRs) [37]. Additionally, it showed a variety of detrimental effects on honey bee behavior, as well as on adult emergence and survival, either alone or in combination with other stressors [38]. Moreover, recent research has also revealed that chronic FPF or/and azoxystrobin fungicide exposure disrupts the gut microbiota of honey bees, increasing opportunistic bacterial pathogens [39]. However, we lack knowledge of the combined effects of FPF, BQCV, and SBV on adult honey bees and of whether these interactions are modulated by a change in the mode of transmission of these viruses (feeding vs. injection).

Here, by using an experimental design with a controlled laboratory, we investigated the effects of the mode of horizontal transmission (feeding vs. injection) of BQCV and SBV alone or in combination with FPF exposure on honey bee survival and transcriptome response by using high-throughput RNA sequencing (RNA-Seq). We used a mixed (BQCV and SBV) viral inoculum, as the two viruses are commonly found to co-occur in honey bees [20]. We hypothesized that the high virulence of BQCV and SBV due to injection (similar to indirect, vector-mediated horizontal transmission) would be explained by stronger immune responses than those found in direct fecal/food–oral transmission and that the host’s response to the infection could be exacerbated by FPF insecticide exposure.

## 2. Materials and Methods

### 2.1. Honey Bees

Three colonies of local *Apis mellifera carnica* that were maintained in the General Zoology apiary at Martin Luther University Halle-Wittenberg, Germany were used from June to August 2020. Before beginning any research activities, colonies were visually checked for *V. destructor* mites and with quantitative real-time PCR (qPCR) for seven common viral targets: acute bee paralysis virus (ABPV), deformed wing virus (DWV) (genotypes A and B), BQCV, chronic bee paralysis virus (CBPV), SBV, and slow bee paralysis virus (SBPV). These targets were detected by using the primers listed in Table S1 and the procedures described in [40]. *Varroa destructor* mites were not seen and viruses were not detected in colonies at a cycle (Cq) of 35, which is a threshold that minimizes the rate of false positives [41].

### 2.2. Pesticide

We used analytical-grade flupyradifurone (Sigma Aldrich, catalog #37050-100MG, Germany). We dissolved FPF in dd H_2_O to obtain stock solutions with a concentration of 1 μg/μL, which were stored at −20 ℃ to avoid degradation. The feeding solutions were prepared by diluting the stock solution with 50 % (*w*/*v*) aqueous sugar (sucrose) solution. The feeding solution was given to the bees ad libitum at the start of the experiment and was replenished every 24 ± 2 h. FPF dilutions were freshly prepared from stock solutions every 2 days. Therefore, the feeding solutions contained either pure sucrose (negative control treatment) or flupyradifurone (FPF treatment; 0.0043 μg FPF μL^−1^). Our dosage was informed by the Environmental Protection Agency’s (EPA) data, which provided residue levels of FPF in nectar and pollen from a variety of plants, such as oilseed rape (4.3 ppm in nectar, which is what we used, and 21.0 ppm in pollen) [42].

### 2.3. Viral Inoculum and Infection

Aliquots of BQCV inoculum were obtained from the propagated inoculum of [43]. We checked the inoculum by using reverse transcription quantitative PCR (qPCR) at the start of the experiment in 2020 with the methods and primers described in Table S1, and we found that it contained only BQCV and no other common honey bee RNA viruses. In 2022, we checked the genetic makeup of the inoculum by extracting RNA from it by using the methods described in [40]. RNA was then submitted to commercial mRNA library preparation for the RNA-Seq analysis by using ultra-deep next-generation sequencing (NGS) on an Illumina platform (GATC Biotech). Surprisingly, the bioinformatic analysis of the NGS library generated from the inoculum revealed that it comprised 96% SBV and 1% BQCV plus 3% honey bee transcripts. It is worth noting that we discovered two new SBV variants in our inoculum, which is why it was not identified in 2020 with the SBV primer pair designed by [44] (Table S1). We named one variant Czech SBV because it was 99% identical to the NCBI entry KY273489, which was sampled from the Czech Republic, and it was found as 56% of the inoculum reads. In the case of the other variant, which we named German SBV, we found no similar NCBI entries to it; the closest entry was KY273489, to which it had 83% sequence similarity. The German SBV variant had a 40% prevalence (NGS reads) in the inoculum (for more details, see the supplementary materials). As a result, we designed a new qPCR primer pair for SBV (Sequence 5’-3’, F: TATGGTGATGATTTAATAATGTC, R: ACAAATGCGGATACACTTC, T_m_ = 50–53 °C) that worked well with our samples in revealing the presence of all SBV variants in our inocula and experimental treatment groups.

The concentrations of BQCV and SBV in the inoculum were then quantified by using qPCR with the BQCV primer pair listed in Table S1 and our new SBV primer pair. We found that our viral inoculum contained 10^8^ and 10^10^ genome equivalents per µL of BQCV and SBV, respectively.

### 2.4. Exposure to FPF Insecticide and Virus

By using a fully crossed laboratory experimental design, honey bees were infected with our BQCV/SBV inoculum either through feeding or through injection, alone or in combination with exposure to FPF pesticide. For each of our three honey bee colonies, we transferred a single frame of sealed worker brood to an incubator kept at 34 °C and 80% relative humidity (RH) overnight. On the next day, we collected 150 newly emerging bees per colony, and then we randomly assigned them to six treatments: control, BQCV/SBV-feeding (virus feeding or VF), BQCV/SBV-injection (virus injection or VI), FPF, VF+FPF (VF+FPF), and VI+FPF (VI+FPF).

For the VF treatment, 10 µL of 50% sugar solution containing viral inoculum was fed to individual bees that had been starved for 2 h, but without prior anesthesia, by using a micropipette. For the VI treatment, bees were chilled on ice for 3 min, and then, one µL of the viral inoculum was injected directly into the hemolymph between a bee’s second and third abdominal tergites by using a Hamilton syringe (the hypodermic needle’s outer diameter: 0.235 mm).

To control for the effects of either injection or feeding during treatments, the control, VI, FPF, and VI+FPF groups were also fed 10 µL of 50% sugar solution devoid of viruses, and, similarly, the control, VF, FPF, and VF+FPF groups were injected with one µL of buffer (PPB) devoid of viruses. We then kept the bees in metal cages (10 × 10 × 6 cm). Each cage contained 25 newly emerged worker bees with the same treatment, which was provided with sugar water ad libitum (control, VF, and VI) or sugar water containing FPF (0.0043 µg µL^−1^) (FPF, VF+FPF, and VI+FPF). The cages were placed in incubators at 30 ± 1 °C and 50% RH. Three replicate cages were used for each treatment. Mortality was recorded daily for 10 days as our measure of the virulence of the viruses (BQCV and SBV) and FPF exposure. Then, we collected subsamples (3 bees per cage, i.e., 9 bees per treatment) at 7 days post-infection and froze them at −80 °C prior to RNA extraction and NGS, as described below.

### 2.5. RNA Extraction and Sequencing

Total RNA was extracted from each individual (whole body, 9 bees per treatment) by using Trizol and the RNeasy Mini Kit (Qiagen, Hilden, Germany). The RNA concentration, purity, and integrity were determined by using an Agilent 4200 TapeStation and six RNA ScreenTapes. Samples that passed our quality criteria (260/280 = 2.1 ± 0.1 and RINe > 9) were used for RNA sequencing. The library preparation from polyA-selected RNA and RNA-Seq was carried out commercially (GENEWIZ Germany GmbH, Leipzig, Germany). Paired-end strand-specific reads of 150 bp in length were obtained by using the Illumina HiSeq platform with approximately 15–20 million read pairs per sample.

### 2.6. RNA-Seq Data Analysis

The datasets were quality-checked, filtered, and trimmed and paired-end reads were merged by using FastP [45]. Then, the cleaned reads were simultaneously mapped to the following genomes by using HISAT2 [45] with a threshold of about 90% genetic similarity: GCF_003254395.2_Amel_HAv3.1_genomic.fna (honey bee genome), the DWV (both variant A and B) assembled from our lab samples [40], the BQCV genome assembled from our inoculum, the Czech SBV assembled from our inoculum, the German SBV assembled from our inoculum, ABPV (NC_002548.1), CBPV (NC_010711.1), IAPV (NC_009025.1), KBV (NC_004807.1), SBPV (NC_014137.1), and Lake Sinai virus (LSV) (KM886905.1). From the resulting SAM files, the contribution of each genome was calculated. Except for the BQCV and SBV strains, none of the samples contained reads that mapped to the genomes of any of the other viruses. Nonetheless, eight samples (VI (3 samples), FPF (2), VI+FPF (2), and VF (1)) were found to be contaminated with DWV-A or DWV-B and were, thus, excluded from further data analysis. One sample from the VI+FPF group was also excluded because the viral infection was unsuccessful (i.e., no reads mapped to BQCV/SBV genomes) (Figure S1).

#### Differential Gene Expression Analysis

Trimmed and filtered RNA-Seq reads were pseudoaligned to the honey bee transcriptome (GCF_003254395.2_Amel_HAv3.1_cds_from_genomic.fna) and simultaneously quantified by using Kallisto v0.46.2 [46]. To analyze the differential gene expression between treatments, we used the tximport package v1.16.1 [47] in R v4.1.2 [48] to import transcript-level read quantification data and convert these into gene-level quantification values (Table S2). We then ran DESeq2 v1.28.1 [49] with the default settings (the adjusted *p*-value was calculated to correct for multiple tests following the Benjamini–Hochberg method [50]) to perform pairwise comparisons of gene expression profiles between different treatments and the control. DEGs were identified as those with an adjusted *p*-value of <0.05 and a log2 (fold-change) of >2.0. We then plotted the gene expression profiles of all individuals—excluding nine individuals, as explained above—in a principal component analysis plot (PCA of variance-stabilized read counts, with genes as columns and samples as rows in the PCA matrix; Table S2). The PCA was performed in the R package ggbiplot v0.55 [51], with ellipse probabilities that were set to the 95% confidence level (ellipse.prob = 0.95).

Differentially expressed genes that were identified with DESeq2 and had log2 (fold-change) >2.0 were selected for functional analysis. Gene ontology (GO) enrichment analysis was performed on this list by using g: Profiler (version: e104_eg51_p15_3922dba) with a database that was updated on 22 April 2019. Biological process functional categories and biological (KEGG) metabolic pathways with an adjusted *p*-value of 0.05 or less (Benjamini–Hochberg FDR method) were then identified [52].

### 2.7. Statistical Analysis

Survival analysis was performed with the R package coxme [53] in R by using mixed-effect Cox proportional hazard models, with ‘cage’ as a nested random effect. The models with ‘cage’ gave a consistently better model fit (lower AIC value) than models without this random effect. Right-censored samples (bees removed at day 7 for the RNA-Seq analysis) were recorded in the dataset and incorporated in the Cox proportional hazard models. We tested different treatments (mode of infection (VF or VI) of BQCV and SBV and/or FPF) with a control that lacked viruses or insecticide. To test for differences between treatments, we performed linear contrasts (Tukey test) of the Cox proportional hazard coefficients (hazard ratios; hereafter, HRs) by using the R package multcomp [54] with Bonferroni correction for multiple comparisons. Data visualizations were performed in GraphPad Prism 7.0 (GraphPad, La Jolla, CA, USA). An adjusted alpha level of 0.05 was used to define significance for all tests.

## 3. Results

### 3.1. Effects on Survival

**Figure 1:**
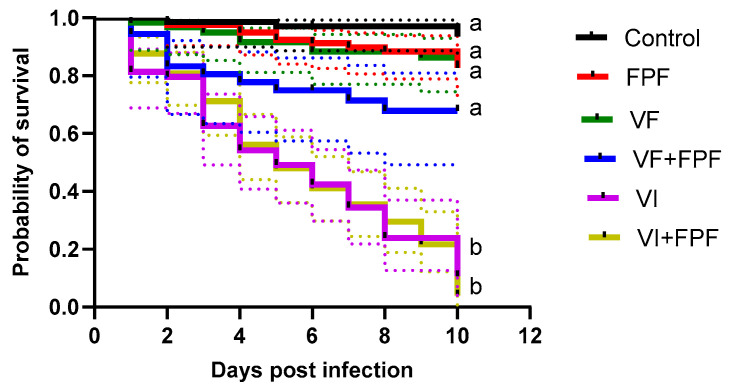
Kaplan–Meier survival curves (solid colored lines) in days post-infection and 95% Cis for each fitted curve (dotted colored lines) of the impact of insecticide (FPF) and RNA viruses (BQCV/SBV) on adult honey bees inoculated orally (VF) or through injection (VI), alone or in combination with FPF. Bees (n = 25 bees per cage, n = 3 cages per treatment) were either injected with one µL inoculum containing 10^8^ and 10^10^ genome equivalents for BQCV and SBV, respectively, or fed 10 µL of sugar syrup containing the same concentrations of viruses on day 0 and subsequently fed with a sublethal (chronic) concentration of FPF (4.3 µg mL^−1^) or a control sugar solution. Pathogens were inoculated once at day 0, while FPF was provided ad libitum throughout the experiment. Different lowercase letters indicate statistically significant differences at α = 0.05 in a Cox proportional hazard mixed-effect model, followed by post hoc Tukey tests (with Bonferroni correction for multiple comparisons). For the statistical details, see Table 1.

Inoculating adult honey bees with BQCV and SBV via injection (VI) significantly reduced their survival regardless of if they were exposed to FPF pesticide (VI+FPF) or not (VI) when compared to controls and those that were orally inoculated, regardless of exposure to FPF (C, VF+FPF, and VF) (*p* < 0.01) (Figure 1, Table 1). There was no significant difference (*p* > 0.05) in survival between the bees that were exposed to FPF (treatment FPF) and orally inoculated (VF, VF+FPF) with BQCV and SBV and the control bees (Figure 1, Table 1).

### 3.2. RNA Sequencing and Mapping Rates

**Figure 2:**
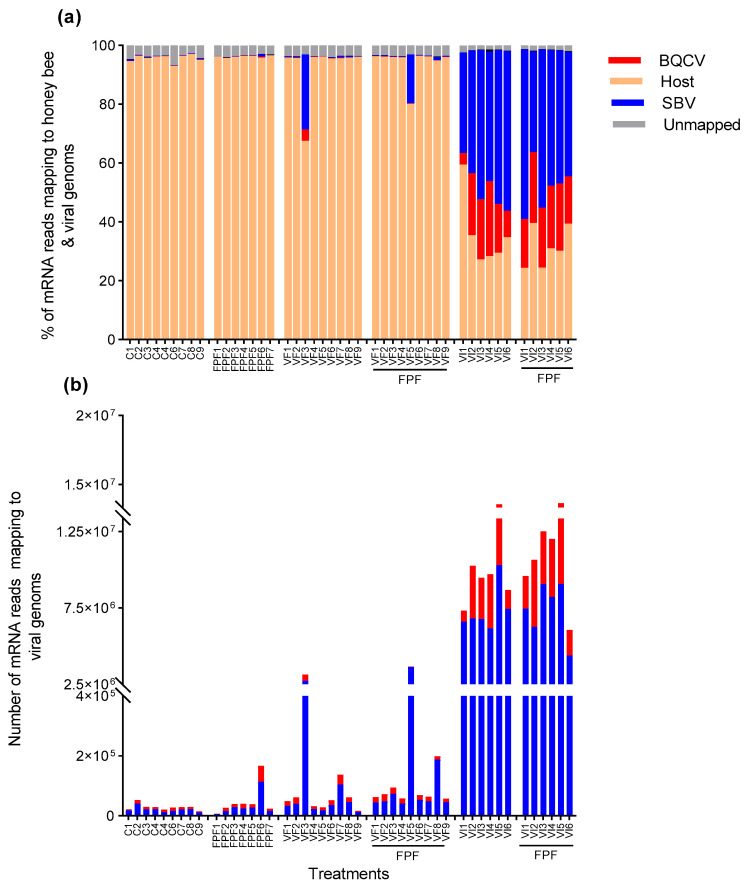
(**a**) Percentage (%) of mRNA reads mapping to the honey bee and viral genomes; (**b**) number of mRNA reads mapping to the BQCV and SBV genomes. Bees (n = 25 bees per cage, n = 3 cages per treatment) were either injected (VI) with one µL of inoculum containing 10^8^ and 10^10^ genome equivalents of BQCV and SBV, respectively, or fed (VF) 10 µL of sugar syrup containing the same concentrations of viruses on day 0 and then fed with a sublethal (chronic) concentration of FPF (4.3 µg mL^−1^) or a control solution for 10 days. Pathogens were inoculated once at day 0, while FPF was fed ad libitum throughout the experiment. Nine bees per treatment were collected at 7 days post-infection. Total RNA was extracted from each bee (whole body); then, paired-end strand-specific reads of 150 bp in length were obtained by using the Illumina HiSeq platform, with approximately 15–20 million read pairs per sample. Datasets were quality-checked, filtered, and trimmed, and the paired-end reads were merged by using FastP. Then, cleaned reads were simultaneously mapped to the honey bee and viral genomes by using HISAT2 with a threshold of 90% genetic similarity. Samples that were found to be contaminated with other viral targets were excluded (see Figure S1).

After trimming and filtering, the sequenced libraries contained, on average, 15 million ± (SD) 4.18 million read pairs (Table S3). Mapping reads to honey bee and viral genomes simultaneously by using HISAT2 revealed that they aligned with the genomes of honey bees, BQCV, and SBV according to the mode of horizontal infection of BQCV and SBV (VF vs. VI). The mean % (±SD) of mRNA reads mapped to SBV and BQCV in bees inoculated via injection (VI) was 46.26 ± 7.61 and 16.07 ± 8.12, respectively; that of those exposed to PFP insecticide (VI+FPF) was 46.60 ± 8.34 and 20.20 ± 3.28, respectively (Figure 2, Table S3).

The percentages of reads mapped to SBV or BQCV did not significantly differ (*p* > 0.05) between the bees that were inoculated via injection and those that were inoculated via injection and exposed to FPF insecticide. Bees that were inoculated orally (VF)—either individually or in combination with exposure to FPF (VF+FPF)—had, on average, 2.63 % ± 6.89 and 0.31 % ± 0.87 of SBV and BQCV reads, respectively. Traces of SBV (0.21% ± 0.16 of reads) and BQCV (0.08 % ± 0.07 of reads) were found in the control bees and those exposed to FPF insecticide alone. That the NGS libraries of all bees in all treatments, including the control (C) and FPF treatments, contained some BQCV and SBV reads possibly reflects a background or latent infection, or it reflects our experimental paradigm in which all bees were injected (with viral inoculum or buffer) and all bees were fed (with viral inoculum and/or FPF or syrup). Uncharacterized/unmapped reads accounted for 3.10 % ± 1.06 of the total reads (Figure 2, Table S3).

### 3.3. Differentially Expressed Genes (DEGs)

**Figure 3:**
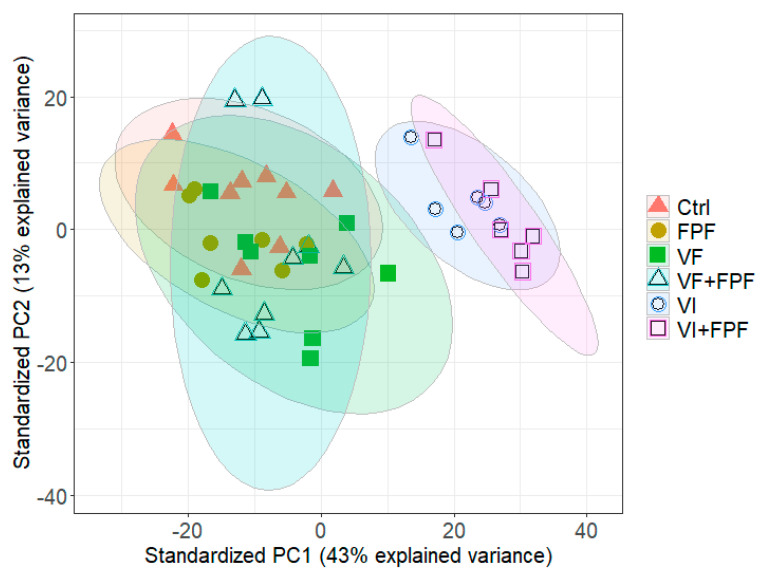
Principal component analysis (PCA) of the variance-stabilized mRNA read counts of the samples used in the final analysis. Bees (n = 6–9 bees per treatment) were either injected with one µL of inoculum containing 10^8^ BQCV and 10^10^ SBV (VI) or fed 10 µL of sugar syrup containing 10^8^ BQCV and 10^10^ SBV (VF), alone or in combination with exposure to the flupyradifuorane (FPF) insecticide (VI+FPF or VF+FPF). The controls and the FPF group received the same treatments, but without a virus. Pathogens were inoculated once at day 0, while FPF was fed ad libitum throughout the experiment. Then, nine bees per treatment were collected at 7 days post-infection. Each point represents the expression profile of a sample (a bee) across all annotated genes (9934). Axis labels give the amount of variance in gene expression explained by the first two principal components (PC1 and PC2). Ellipses represent 95%confidence levels, and colors further illustrate the different treatments.

We compared the gene expression levels based on read counts per gene between the control and treated groups (FPF, VF, VI, VF+FPF, and VI+FPF) for a total of 9934 genes. A PCA of the normalized read counts across all of these genes across the different treatments showed a clear distinction between the gene expression profiles of bees that were inoculated via injection (VI)—whether or not they were exposed to FPF insecticide (VI+FPF, VI)—across both principal component 1 (PC1 explained 43% of the variance) and principal component 2 (PC2 explained 13% of the variance) (Figure 3).

**Figure 4:**
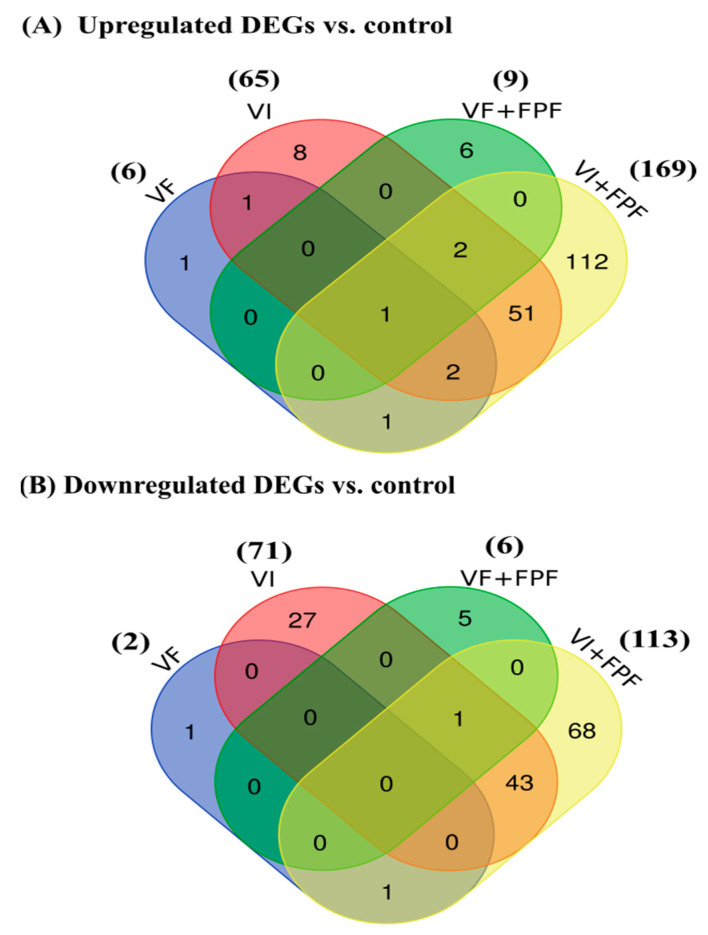
Venn diagram showing the number of significantly differentially upregulated (**A**) and downregulated (**B**) gene transcripts between the control and experimental treatments and the overlap between the different experimental inoculation treatments with the two honey bee viral pathogens (SBV and BQCV) and flupyradifuroane (FPF) insecticide. Bees (n = 6–9 bees per treatment) were either injected with one µL of inoculum containing 10^8^ BQCV and 10^10^ SBV (VI) or fed 10 µL of sugar syrup containing 10^8^ BQCV and 10^10^ SBV (VF), alone or in combination with exposure to flupyradifuorane (FPF) insecticide (VI+FPF or VF+FPF). The controls and FPF group received the same treatments, but without a virus. Pathogens were inoculated once at day 0, while FPF was fed chronically ad libitum throughout the experiment; nine bees per treatment were collected at day 7 post-infection. Differentially expressed genes (DEGs) were identified as those with an adjusted *p*-value of <0.05 and a log2 (fold-change) of >2.0.

DEGs were identified as those with an adjusted *p*-value of <0.05 and a log2 (fold-change) of >2.0. The number of genes exhibiting significant changes in expression level as a result of the mode of infection and/or FPF exposure varied between treatments. In comparison with the control, in bees infected with BQCV and SBV by feeding (VF), 8 genes (6 upregulated and 2 downregulated) were differentially expressed, while 136 genes (65 upregulated and 71 downregulated) were differentially expressed in bees infected via injection (VI) (Figure 4, Supplementary File S1). When bees with double-treatments (VF+ FPF or VI+FPF) were compared to the control bees, we found differential expression of 15 (9 upregulated and 6 downregulated) and 282 (169 upregulated and 113 downregulated) genes, respectively (Figure 4, Supplementary File S1). This suggests that molecular changes occurred in response to the mode of infection with BQCV and SBV (feeding vs. injection), regardless of FPF insecticide exposure. There were no DEGs in bees exposed to FPF insecticide compared to the control group (Supplementary File S1).

### 3.4. Functional Annotation and Classification of the DEGs

Several immunity-related genes were differentially upregulated across the treatments, including three antimicrobial peptide genes (AMPs) (apidaecin 1, hymenoptaecin, and abaecin), three genes related to Toll/TLR (SP34, toll-like receptor Tollo (LOC410235), and serine proteinase stubble (transcript variant X4)), and two genes related to the RNA interface (RNAi) (protein argonaute-2 (AGO2) and endoribonuclease Dicer (transcript variant X1)) (Table 2). The expression of Dicer-like and Argonaute-2 genes, which was quantified by using qPCR in previous studies under the same experimental conditions [18,34], was also consistent with the current study’s RNAseq data (Figure S2). Other genes that were differentially upregulated included two odorant receptor (OR) genes (odorant receptors 13a and Or12), a general odorant-binding protein (OBP) gene (OBP9), a gustatory receptor (GR) gene (gustatory receptor for sugar taste 64f, transcript variant X3), four genes related to the signaling receptor activity of octopamine, serotonin, and nicotinic acetylcholine, two genes related the detoxification of xenobiotics (cytochrome P450 307a1 and cytochrome P450 6A1 (transcript variant X1)), and one gene related to melatonin (melatonin receptor type 1B-B-like) (Table 2).

Several genes, on the other hand, were downregulated in the different treatments, including five genes related to OBP (OBP3, OBP13, OBP14, OBP17, and OBP21), one gene related to ORs (odorant receptor 67a-like (transcript variant X2)), two genes related to eicosanoid biosynthesis (PLA2 and phospholipase A2-like), and five genes involved in lipid transport, localization, and metabolism (LOC409187, PLA2, phospholipase A2 like, vitellogenin, and acyl-CoA Delta (11) desaturase (transcript variant X2)) (Table 3). Other genes involved in the negative regulation of molecular function, modulation of cell killing, hemolysis, proteolysis, cation transmembrane transport, and cytolysis, as well as the myeloid leukocyte and mast cell activation involved in immune response (MCDP, apamin, COX3, chymotrypsin-1, secapin, and Melt), were also differentially downregulated (Table 3). It is noteworthy that ten honey bee venom peptide (HBVP)-encoding genes (apamin, serine protease 53, Pla2-like, Pla2, secapin, venom allergen Api m 6, Melt, Mcdp, phospholipase A1 member A, transcript variant X3) were differentially downregulated in the bees inoculated with BQCV+SBV via injection, regardless of PFP insecticide exposure (Table 3).

Other genes that were either differentially upregulated (Y-e3, CPR1, LOC107964791, LOC100578352, and LOC725629) (Table 2) or downregulated (Est-6, Eth, LOC406114, LOC100576797, and LOC408603) (Table 3) in response to BQCV and SBV infection, whether or not the bees were exposed to FPF pesticide (VI+FPF or VI), were identified. These genes can be considered as candidates for any serious viral infection.

The functional analysis revealed significantly overrepresented GO terms involved in carbohydrate metabolism (Table 4) due to a differentially upregulated gene (LOC113218760: probable galactose-1-phosphate uridylyltransferase (GalT)) shared by all bees that were either injected or fed with a virus alone or in combination with exposure to FPF (Figure 4A). The significantly overrepresented GO terms involved in immunity and defense mechanisms were due to two differentially upregulated genes, apidaecin (Apid1) and hymenoptaecin (LOC406142), that were shared by the VI, VI+FPF, and VF+FPF treatment groups (Table 4). Functional analysis of the six distinct differentially expressed genes in the VF+FPF group revealed an overrepresentation of GO terms related to immune and defensive response, as well as caste determination, which was likely due to the upregulation of the major royal jelly protein 1 (MRJP1) (Table 4). The functional enrichment of the differentially downregulated genes shared by VI and VI+FPF (Figure 4B) revealed that these were mainly involved in lipid transport, localization, metabolism, and fatty acid secretion (Table 5). Full tables of the functional enrichment results are provided in Supplementary File S2.

## 4. Discussion

The shift in the mode of experimental horizontal transmission of BQCV and SBV from fecal/food–oral (direct horizontal) to vector-mediated (indirect horizontal) transmission resulted in a significant increase in mortality and viral titer in adult honey bees, as previously noted in pupae [20] and adult honey bees [19], thus posing a future threat to bees and apiculture. Adult bees that were fed with BQCV and SBV, on the other hand, did not show increased mortality with inoculation, as was also previously observed [19,55,56].

A strength of our experimental paradigm—in which all bees, including those with the control treatment, were injected (either with a viral inoculum or a buffer) and all bees were fed (with a viral inoculum and with FPF or syrup)—is that changes in gene expression could be directly attributed to the mode of viral infection, namely, injection (simulating the vector-borne route of horizontal transmission) or feeding (simulating the fecal–oral route of horizontal transmission). A caveat of this approach is that injection may, per se, induce the replication of a background or latent viral infection. In our case, this may not have allowed us to unambiguously assess the impact of viral feeding on gene expression because the control and viral-fed treatments differed little in terms of the viral titer. Future studies that employ a similar experimental paradigm to ours should consider using an additional control treatment that is not injected (with a buffer) to assess the impact of injection per se on viral replication and gene expression.

The high virulence of BQCV and SBV due to injection (similar to that of indirect, vector-mediated horizontal transmission) found in this and previous research could lead to a stronger immune response than that induced by direct fecal/food–oral transmission, which could also be modulated by FPF insecticide exposure. Our findings support this hypothesis, even though co-exposure to viruses via feeding or injection and FPF insecticide had no interactive effects on their survival. Our RNA-Seq analysis revealed a distinct difference in the gene expression profiles of bees inoculated with viruses via injection (VI) versus feeding (VF), though exposure to FPF insecticide made little overall difference in gene expression, as demonstrated by the PCA. Indeed, the number of genes with significant changes in expression level in bees injected with viruses alone (136) or in combination with FPF insecticide (282) was very high when compared to that in bees that were inoculated with viruses by feeding (8) and/or were exposed to FPF insecticide (15). These transcriptional changes and their associated molecular pathways may underpin the shift in the experimental transmission of BQCV and SBV that led to a higher rate of mortality in both the current study and previous research [18,19,34].

GalT is an enzyme involved in galactose metabolism via the Leloir pathway [57]. In humans, severe GalT impairment results in the potentially fatal condition known as classic galactosemia [58]. In the current study, GalT was significantly upregulated in VI and VF bees, regardless of FPF exposure. The functional annotation of GalT revealed that the GO terms involved in carbohydrate metabolism were significantly overrepresented. This suggests that increased energy metabolism may be required to mount an immune defense against viral infection, as noted in response to DWV infection [59].

The VI, VI+FPF, and VF+FPF treatment groups all shared the AMP genes apidaecin (Apid1) and hymenoptaecin, which caused GO terms involved in immunity and defense mechanisms to be significantly overrepresented. Abaecin, another AMP gene, was found to be upregulated in only the VI bees. Other genes associated with immunity that were only shared by the bees in the VI and VI+FPF groups included two RNAi-related genes: the protein argonaute-2 (AGO2) and the endoribonuclease Dicer (transcript variant X1).

RNAi is the primary antiviral defense mechanism in insects [60,61,62], though AMPs have also been shown to be activated during viral infection [63,64]. Induction of RNAi components and AMP-related genes has also been observed in honey bees in response to infection with DWV genotypes A and B, IAPV, SBV, and BQCV [19,34,63,65,66,67], indicating that bees mount a response to viral challenges, though it is difficult to determine whether this response is successful in limiting viral proliferation or not. Three Toll/TLR-related genes—SP34, Toll-like receptor Tollo (LOC410235), and serine proteinase stubble (transcript variant X4)—were also involved in the immune responses of only the VI+FPF bees. The Toll pathway has previously been shown to be involved in defense against viral invasion in insects [65,68,69]; however, it is unclear why these Toll/TLR-related genes were only activated in the VI+FPF bees and not in the VI-bees in our study.

Insects recognize odor molecules in their external environment by using a variety of proteins that are involved in the olfactory recognition system, including odorant binding proteins (OBPs), chemosensory proteins, and chemoreceptors [70]. Previous research has demonstrated that the downregulation of OBP genes in bees exposed to neonicotinoid insecticides may have an impact on their chemosensory abilities, thus impairing olfactory associative functions, such as foraging behaviors, olfactory learning, and honey bee memory [71,72,73]. In the current study, the differentially downregulated genes in the VI and VI+FPF bees were found to be enriched in GO terms associated with lipid transport, localization, metabolism, and fatty acid secretion. Five of these genes were associated with OBP—OBP3, OBP13, OBP14, OBP17, and OBP21—while the other two were associated with odorant receptors (ORs) and chemosensation (CSP6), though some OBPs and gustatory receptors (GRs) were found to be upregulated as well. This suggests that the shift in the experimental transmission of BQCV and SBV could alter olfactory associative functions, such as foraging behaviors, olfactory learning, and honey bee memory. These findings may also explain the specific learning deficits observed in honey bee foragers inoculated with DWV or IAPV via injection (increased responsiveness to water and low sucrose concentrations and impaired associative learning, memory formation, and homing ability) [74,75]. Furthermore, CBPV-infected bees suffer from hive mate nibbling attacks, resulting in hair loss [76], indicating that bees possess the sensory capacity to detect virus-infected bees. The potential downregulation of sensory functions found in the current study due to the shift in the mode of transmission may also have negative consequences for bees’ social hygienic behavior.

Honey bee venom peptides (HBVPs) have been shown to contribute to the social immunity of bees [77]. In the current study, ten HBVP-encoding genes (apamin, serine protease 53, Pla2-like, Pla2, secapin, venom allergen Api m 6, Melt, Mcdp, phospholipase A1 member A, transcript variant X3) were found to be differentially downregulated in bees inoculated with BQCV and SBV via injection regardless of FPF insecticide exposure. The Pla2 and Pla2-like genes are involved in the eicosanoid biosynthesis pathway, which is part of the immune system of insects, influences several aspects of cellular immunity, and has been linked to antiviral immune responses [69,78]. Toll/IMD signaling is also linked to eicosanoid signaling; Toll and IMD activate PLA2, which leads to eicosanoid biosynthesis [79]. In addition, melittin belongs to the class of bee-venom-derived antiviral peptides (AVPs) and was isolated from the honey bee *A. mellifera* [80]. This AVP was also tested against a wide range of viral pathogens, and it inhibited viral replication for all viruses tested [81]. Secapin-1 is also a multi-functional HBVP with anti-fibrinolytic, anti-elastolytic, and anti-microbial properties. Secapin-1 transcript expression was found to be significantly higher in the fat bodies of *A. cerana* worker bees injected with bacteria and fungi, indicating that Secapin-1 plays a role in innate immunity in response to microbial infection [82]. Similarly, the allergen Api m 6-like HBVP functions as a serine protease inhibitor with antibacterial and antifungal properties [83], implying its role in innate immunity in response to microbial infection. Surprisingly, mast cell degranulating peptide (Mcdp), a neuro- and immunotoxic HBVP known for its degranulating effect on vertebrate granulocytes [84,85], was highly downregulated in the current study. Mcdp was also differentially expressed in immune-primed bumblebee workers but not in directly bacterially challenged workers [86], suggesting that it plays a role in the innate immune response of bees. Given the importance of HBVPs in honey bee immunity, the inhibition of HBVP-encoding genes as a result of a change in the viral mode of infection could be the cause of the high virulence of BQCV and SBV observed in this and previous research [18,19,34].

Apidermin-3 plays a role in innate immune responses in honey bees [87]. Apidermin-3 is a cuticular protein that has recently been implicated as an outlier protein that is suppressed by DWV injection in varroa-mite-resistant honey bee stock [88,89] and was found in response to BQCV and SBV injection and FPF insecticide exposure in the current study. In addition, vitellogenin, which is a reproduction- and nutrition-related MRJP and has also been linked to immunity in honey bees [90,91], was downregulated in the VI and VI+FPF bees in the present study. Similarly, a vitellogenin precursor was found to be downregulated in DWV-infected and varroa-mite-parasitized bees [88]. A decrease in vitellogenin was linked to hemocyte apoptosis; this mechanism was described for worker bees, which dramatically downregulated their defense machinery when they transitioned from hive workers into foragers [92]. This suggests that BQCV and SBV suppress vitellogenin to prevent viral destruction via hemolymph-based immune protection, as was also reported for DWV infection [88].

Pathogens can alter a host’s physiology and metabolism, influencing several important life-history traits and altering functions involved in pesticide toxicokinetics and toxicodynamics, such as in the detoxification system [35]. When we investigated whether the FPF insecticide affected the transcriptional reactions of bees to the altered mode of viral transmission, we discovered that the VF+FPF and VI+FPF bees had more unique DEGs than the VF and VI bees did, respectively. These genes included the multifunctional proteins MRJP1, 5, and 6, which were expressed in the VF+FPF bees. MRJPs were induced in DWV-infected and varroa-mite-parasitized bees [88]. The higher content of MRJPs was likely related to immune defense [93]. Some detoxification-encoding genes (cytochrome P450 307a, cytochrome P450 6A1, and transcript variant X1) were induced in the VI+FPF bees, whereas others, such as cytochrome P450 genes (cytochrome P450 9e2 and probable cytochrome P450 6a13), were suppressed in the bees in the VI+FPF treatment group. This suggests that BQCV and SBV injection could reduce bees’ detoxification capacity, but, surprisingly, these changes did not result in decreased survival of bees treated with FPF, BQCV, and SBV (compared to the virus alone), as was previously reported [34].

Given that SBV was at a higher titer than BQCV in the inoculum used in the current study, which resulted in differences in viral load, as shown in Figure 2, one might assume that the observed transcriptional changes were mainly caused by SBV rather than BQCV. However, viral co-infections are common in insects, and both may cause a change in gene expression. Following experimental DWV infection, for example, BQCV and SBV loads concurrently increased [20]. There is also evidence of co-prevalence between these two viruses [30]. Despite the absence of studies focusing on BQCV–SBV interactions, their shared infection patterns and correlated prevalence suggest that they may both impact gene expression; they warrant further investigation, which is critical in anticipating their future evolution and management. The understanding of the interactive effects of BQCV and SBV on hosts would also benefit from studies of the impact on hosts of each virus—BQCV and SBV—in isolation. This would require the generation of pure inocula or the use of a viral clone that can guarantee that an inoculum contains only a single virus.

Finally, it is unclear why only one replicate of each of the two feeding treatments (VF3 and VF5+FPF) was positive or significantly different from those of the other bees with the VF/VF+FPF treatments, respectively. To partly address this issue, future VF experiments could compare infection in treated bees with infection in bees that have been orally inoculated with an inactivated viral stock so as to check whether or not the few infected bees represent a background infection in the hosts. Despite this shortcoming, the profound drop in host survival and change in gene expression following viral transmission via injection (simulating vector-mediated transmission) compared to feeding (fecal–oral route) is unambiguous.

## 5. Conclusions

A shift in the mode of experimental transmission of BQCV and SBV from fecal/food–oral (direct horizontal) to vector-mediated (indirect horizontal) transmission resulted in a significant increase in mortality and viral titer in adult honey bees. The transcriptomic analysis provided plausible explanations for the increased virulence of BQCV and SBV. Several immune-related genes were upregulated in bees that acquired viruses via injection, either alone or in combination with FPF insecticide, while several OBPs, chemosensory receptors, and odor receptors, as well as ten HBVPs and an MRJP (vitellogenin)-encoding gene, were suppressed. Responses to sublethal doses of insecticide may not lead to a change in mortality (by definition, they are sublethal), but they nevertheless lead to marked changes in gene regulation, as we found in the current study. Therefore, we may be underestimating the impacts of pesticides on bee fitness when using superficial assays, such as that of mortality. This study provides the first insight into the molecular mechanisms underlying the high virulence of BQCV and SBV due to an experimental change in the mode of transmission, and candidate genes that may respond to any viral infection were identified.

## Figures and Tables

**Table 1:** Impact of the mode of transmission of viruses and/or pesticide treatment on honey bee survival based on Cox proportional hazard models; model-averaged β coefficients (standardized effect size of the hazard, where higher β indicates a higher risk of death) of two horizontal modes of transmission of BQCV/SBV (feeding (VF) vs. injection (VI)) and flupyradifurone (FPF) insecticide either alone or in combination. The exp. β is equivalent to the hazard ratio obtained from a Cox proportional hazard model with respect to the control. Treatments with two factors were also compared to treatments with a single factor. In bold are the treatments that were significant according to *post hoc* Tukey tests (with Bonferroni correction for multiple comparisons).

Variables	β	SE of β Coefficient (+/−)	Exp. β	Z	*p*
FPF	1.05	0.64	2.87	1.64	1.00
VF	0.91	0.68	2.49	1.34	1.00
VF+FPF	1.95	0.67	7.03	2.91	0.05
VI	3.61	0.60	37.09	5.97	**<0.001**
VI+FPF	3.68	0.60	39.68	6.12	**<0.001**
**Interaction between viruses and FPF**					
VF+FPF vs.			VF		1.00
			FPF		1.00
VI+FPF vs.			VI		1.00
			FPF		**<0.001**

**Table 2:** Upregulated differentially expressed genes.

Gene Name	Gene Description	Category	(log2 Fold Change)			
			VF	VF+FPF	VI	VI+FPF
**LOC113218760**	probable galactose-1-phosphate uridylyltransferase	Carbohydrate metabolism	5.46	5.02	6.28	6.32
**LOC725754**	zinc finger protein castor homolog 1, transcript variant X4	Cell communication and signaling	2.15	-	2.76	2.44
**Oa1**	octopamine receptor	Signaling receptor activity	2.41	-	-	-
**Mrjp1**	major royal jelly protein 1	MRJP family	-	2.36	-	-
**Mrjp5**	major royal jelly protein 5	MRJP family	-	3.38	-	-
**Mrjp6**	major royal jelly protein 6	MRJP family	-	2.60	-	-
**Apid1**	apidaecin 1	Antimicrobial peptides (AMPs)	-	2.4	3.71	3.8
**LOC406142**	hymenoptaecin	AMPs	-	2.21	2.68	2.93
**LOC411577**	protein argonaute-2	RNAi	-	-	2.72	2.6
**LOC726766**	endoribonuclease Dicer, transcript variant X1	RNAi	-	-	2.17	2
**Y-e3**	yellow-e3	MRJP family	-	-	2.35	2.85
**CPR1**	cuticular protein 1	Structural constituent of cuticle	-	-	2.56	3.19
**nAChRb2**	nicotinic acetylcholine receptor beta2 subunit	Signaling receptor activity	-	-	2.83	2.56
**LOC107964791**	titin-like	Hypertrophic cardiomyopathy (HCM)	-	-	2.62	3.42
**Obp9**	odorant binding protein 9	Odorant receptor	-	-	3.6	4.25
**LOC406144**	abaecin	AMPs	-	-	2.26	-
**nAChRa9**	nicotinic acetylcholine receptor alpha9 subunit	Signaling receptor activity	-	-	2.12	-
**LOC100578352**	ionotropic receptor 75a-like		-	-	3.19	3.36
**LOC725629**	DNA-binding protein D-ETS-6-like		-	-	4.50	4.03
**5-ht7**	serotonin receptor 7	Signaling receptor activity	-	-	-	2.42
**CPR17**	cuticular protein 17	Structural constituent of cuticle	-	-	-	3.12
**LOC100576212**	odorant receptor 13a	Odorant receptor	-	-	-	2.97
**LOC410235**	toll-like receptor Tollo	Toll	-	-	-	2.43
**LOC410495**	cytochrome P450 307a1	Detoxification	-	-	-	2.69
**LOC413908**	cytochrome P450 6A1, transcript variant X1	Detoxification	-	-	-	5.71
**LOC727431**	gustatory receptor for sugar taste 64f, transcript variant X3	chemosensation	-	-	-	2.36
**Or12**	odorant receptor 12	Odorant receptor	-	-	-	2.34
**SP34**	serine protease 34	Toll/TLR	-	-	-	3.83
**TyHyd**	tyrosine hydroxylase	Tyrosine metabolism	-	-	-	2.04
**LOC107964335**	melatonin receptor type 1B-B-like	Signaling receptor activity	-	-	-	5.14
**LOC410624**	serine proteinase stubble, transcript variant X4	Toll/TLR	-	-	-	2.81

**Table 3:** Downregulated differentially expressed genes.

Gene Name	Gene Description	Category	(log2 Fold Change)			
			VF	VF+FPF	VI	VI+FPF
**Obp13**	odorant binding protein 13	odorant-binding protein (OBP)	-	−2.35	−2.85	−2.33
**Apamin**	apamin protein	-	-	-	−5.51	−5.64
**Apd-3**	apidermin 3	innate immune	-	-	−2.95	−2.25
**Est-6**	venom carboxylesterase-6	-	-	-	−2.30	-
**Eth**	ecdysis triggering hormone	-	-	-	−2.71	−2.26
**LOC102653899**	probable cytochrome P450 6a13	-	-	-	−3.59	−3.05
**LOC102654530**	odorant receptor 67a-like, transcript variant X2	Odorant receptor (OR)	-	-	−2.43	-
**LOC724308**	serine protease 53	Toll/TLR	-	-	−2.14	−2.58
**LOC724436**	phospholipase A2-like	Eicosanoid	-	-	−4.63	−3.76
**Pla2**	phospholipase A2	Eicosanoid	-	-	−5.82	−6.47
**Vg**	vitellogenin	vitellogenin	-	-	−3.29	−2.99
**LOC406145**	secapin	innate immune response	-	-	−6.10	−5.33
**LOC678674**	venom allergen Api m 6	-	-	-	−5.27	−7.50
**LOC406114**	alpha-amylase	-	-	-	−3.44	−4.00
**Melt**	melittin	innate immune	-	-	−5.46	−4.43
**LOC100576797**	acyl-CoA Delta (11) desaturase, transcript variant X2	lipid metabolism	-	-	−5.21	−4.89
**Obp17**	odorant binding protein 17	OBP	-	-	−2.56	−3.02
**Obp3**	odorant binding protein 3	OBP	-	-	−2.41	−3.50
**LOC724175**	probable cytochrome P450 304a1	-	-	-	−2.81	−3.13
**Mcdp**	mast cell-degranulating peptide	-	-	-	−6.30	−6.13
**LOC408603**	glucose dehydrogenase [FAD, quinone]	-	-	-	−2.96	−4.68
**LOC727037**	phospholipase A1 member A, transcript variant X3	Eicosanoid	-	-	−2.34	−2.73
**Obp14**	odorant binding protein 14	OBP	-	-	−3.71	−2.82
**COX3**	Cytochrome c oxidase subunit 3	mitochondrial energy metabolism	-	-	-	−2.16
**CSP6**	chemosensory protein 6		-	-	-	−2.22
**Obp21**	odorant binding protein 21	OBP	-	-	-	−2.69
**LOC725922**	mitochondrial basic amino acids transporter	Transport	-	-	-	−2.88
**LOC724211**	cytochrome P450 9e2	-	-	-	-	−2.31
**LOC551197**	probable cytochrome P450 6a13	-	-	-	-	−2.03
**LOC410894**	chymotrypsin-1	proteolysis	-	-	-	−2.21
**LOC411307**	mitochondrial enolase superfamily member 1	cellular amino acid catabolic process	-	-	-	−2.02

**Table 4:** Top ten biological processes that were enriched in significantly upregulated and differentially expressed genes (DEGs) in—or overlapping across—the treatment groups (for more details, see Figure 2a).

**GO: Category**	**GO: Biological Process Term**	***p* Value ^1^**	**DEGs ^2^**	**Category ^3^**	**Treatment**
GO:0019320	Hexose catabolic process	1.37 × 10^−3^	1	3	VI, VF, VI+FPF, VF+FPF
GO:0019388	Galactose catabolic process	1.37 × 10^−3^	1	3	
GO:0033499	Galactose catabolic process via UDP-galactose	1.37 × 10^−3^	1	3	
GO:0046365	Monosaccharide catabolic process	1.37 × 10^−3^	1	4	
GO:0006012	Galactose metabolic process	2.74 × 10^−3^	1	10	
GO:0005996	Monosaccharide metabolic process	6.17 × 10^−3^	1	36	
GO:0016052	Carbohydrate catabolic process	6.17 × 10^−3^	1	27	
GO:0019318	Hexose metabolic process	6.17 × 10^−3^	1	34	
GO:0044282	Small molecule catabolic process	7.62 × 10^−3^	1	50	
GO:0005975	Carbohydrate metabolic process	2.15 × 10^−2^	1	157	
GO:0009617	Response to bacterium	3.51 × 10^−6^	2	9	VI, VF+FPF, VI+FPF
GO:0042742	Defense response to bacterium	3.51 × 10^−6^	2	9	
GO:0006952	Defense response	5.39 × 10^−6^	2	22	
GO:0006955	Immune response	5.39 × 10^−6^	2	24	
GO:0009607	Response to biotic stimulus	5.39 × 10^−6^	2	21	
GO:0043207	Response to external biotic stimulus	5.39 × 10^−6^	2	21	
GO:0044419	Biological process involved in interspecies interaction between organisms	5.39 × 10^−6^	2	24	
GO:0045087	Innate immune response	5.39 × 10^−6^	2	17	
GO:0051707	Response to other organism	5.39 × 10^−6^	2	21	
GO:0098542	Defense response to other organism	5.39 × 10^−6^	2	21	
GO:0050896	Response to stimulus	5.67 × 10^−3^	2	902	
GO:0050832	Defense response to fungus	1.81 × 10^−3^	1	1	VF+FPF
GO:0050830	Defense response to Gram-positive bacterium	1.81 × 10^−3^	1	1	
GO:0050829	Defense response to Gram-negative bacterium	1.81 × 10^−3^	1	1	
GO:0048651	Polyphenic determination, influence by environmental factors	1.81 × 10^−3^	1	1	
GO:0048650	Caste determination, influence by environmental factors	1.81 × 10^−3^	1	1	
GO:0009620	Response to fungus	1.81 × 10^−3^	1	1	
GO:0048648	Caste determination	1.81 × 10^−3^	1	1	
GO:0048647	Polyphenic determination	1.81 × 10^−3^	1	1	
GO:0001906	Cell killing	2.90 × 10^−3^	1	2	
GO:0031640	Killing of cells of other organisms	2.90 × 10^−3^	1	2	

^1^ Overrepresented *p*-value for this category. ^2^ Number of differentially expressed genes in this category identified by g: Profiler with a cutoff of *p* < 0.05. ^3^ Total number of genes in this biological process category.

**Table 5:** Top ten biological processes that were enriched in significantly downregulated differentially expressed genes (DEGs) overlapping across treatment groups (for more details, see Figure 2b).

**GO: Category**	**GO: Biological Process Term**	***p* Value ^1^**	**DEG ^2^**	**Category ^3^**	**Treatment**
GO:0035821	Modulation of the processes of other organisms	2.96 × 10^−5^	3	4	VI, VI+FPF
GO:0055114	Obsolete oxidation–reduction process	1.55 × 10^−3^	8	334	
GO:0010876	Lipid localization	1.93 × 10^−3^	4	56	
GO:0006869	Lipid transport	1.93 × 10^−3^	4	54	
GO:0044419	Biological process involved in interspecies interaction between organisms	2.86 × 10^−3^	3	24	
GO:0015909	Long-chain fatty acid transport	4.23 × 10^−3^	2	7	
GO:0071715	Icosanoid transport	4.23 × 10^−3^	2	7	
GO:0050482	Arachidonic acid secretion	4.23 × 10^−3^	2	7	
GO:0044255	Cellular lipid metabolic process	4.23 × 10^−3^	5	167	
GO:0032309	Icosanoid secretion	4.23 × 10^−3^	2	7	

^1^ Overrepresented *p*-value for this category. ^2^ Number of differentially expressed genes in this category identified by g: Profiler with a cutoff of *p* < 0.05. ^3^ Total number of genes in this biological process category.

## Data Availability

The data presented in this study are available in the Supplementary Materials.

## Supplementary Materials

The following supporting information can be downloaded at: https://www.mdpi.com/article/10.3390/v15061284/s1, Figure S1: mRNA read counts mapping to honey bee and viral genomes; Figure S2: Expression of two genes (Dicer-like and Argonaute-2) as quantified via qPCR in previous research that followed the same experimental conditions and via the RNA-Seq data in the current study. Table S1: Primers used for the quantification of viruses by using reverse transcription followed by qPCR and the efficiency of qPCR. Table S2: DESeq2 count matrix; Table S3: Information about the analyzed RNA-Seq samples. Supplementary file S1: DESeq2 results for each pairwise comparison with the control and the likelihood-ratio test. Supplementary file S2: Functional enrichment of genes that were differentially expressed in the different treatments.
