# Supplementary material for: Transcriptomic Responses Underlying the High Virulence of Black Queen Cell Virus and Sacbrood Virus following a Change in Their Mode of Transmission in Honey Bees (Apis mellifera)
Source: Viruses. 2023 May 30;15(6):1284. doi: 10.3390/v15061284 (PMC10303220; doi:10.3390/v15061284)
Supplement: Supplementary file 1 [file viruses-15-01284-s001.zip › Supplementary materials_RJP.pdf]

Supplementary Materials

Assembling sac brood virus (SBV) genome from start inoculum

We used the NGS library from the BQCV start inoculum, which had 75 million paired-end reads (2 x 150 nucleotides), to assemble the SBV genome because it contained a significant amount of SBV reads. The NGS library is trimmed for low-quality bases, and the overlapped paired reads are merged using fastp [1]. The resulting reads were mapped to a collection of genomic data including honey bee genome, honey bee transcriptome, and BQCV genome using hisat2 [2] with the default settings, yielding reads with at least 95% genetic similarity as mapped (roughly). The unmapped reads were extracted and assembled using rnaviralspades [3] with default setting; subsequently the resulting scaffolds with a minimum length of 7000 nucleotides and a minimum K-mer coverage of 10000 were extracted and blasted against the NCBI nucleotide collection using NCBI online tools to identify the scaffolds. From NCBI blasting results, we found three variants of SBV: 1. Czech SBV which is 99% similar to the NCBI entry KY273489, sampled from Czech republic, and contributes 60% of SBV in the start inoculum. 2. Swedish SBV which is 99.7% similar to MT636331, collected from Sweden. There is only a trace of this variant in start inoculum. 3. German SBV: We didn’t find any similar entry from NCBI for this variant. The closest one is KY273489 which is 83% similar. This sequence contributes to SBV in the start inoculum by 40%.

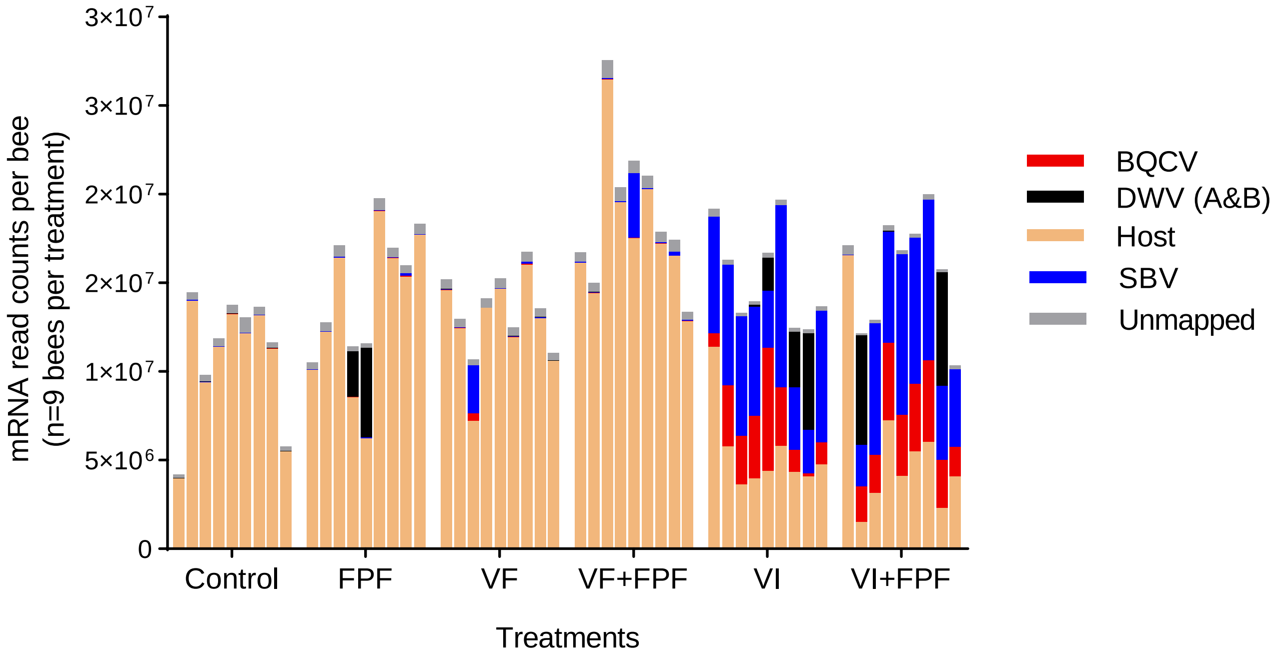

Figure S1. mRNA reads counts mapping to honey bee and viral genomes. Bees (n = 25 bees per cage, n = 3 cages per treatment) were either injected (VI) with one µL inoculum containing 10^8 and 10^{10} genome equivalents of BQCV and SBV, respectively, or fed (VF) 10 µL of sugar syrup containing the same concentrations of viruses on day 0 and then fed with a sublethal concentration of FPF (4.300 µg ml^{-1}) or a control solution for 10 days. Pathogens were inoculated once at day 0 while FPF was fed *ad libitum* across the experiment. Nine bees per treatment were collected at 7 days post infection. Total RNA was extracted from each bee (whole body) then paired-end strand-specific reads of 150 bp in length were obtained using the Illumina HiSeq platform, with approximately 15-20 million read pairs per sample. Datasets were quality-checked, filtered, trimmed, and pair end reads merged using FastP, then cleaned reads were mapped to honey bee and viral genomes simultaneously using HISAT2 with a threshold of 90% genetic similarity.

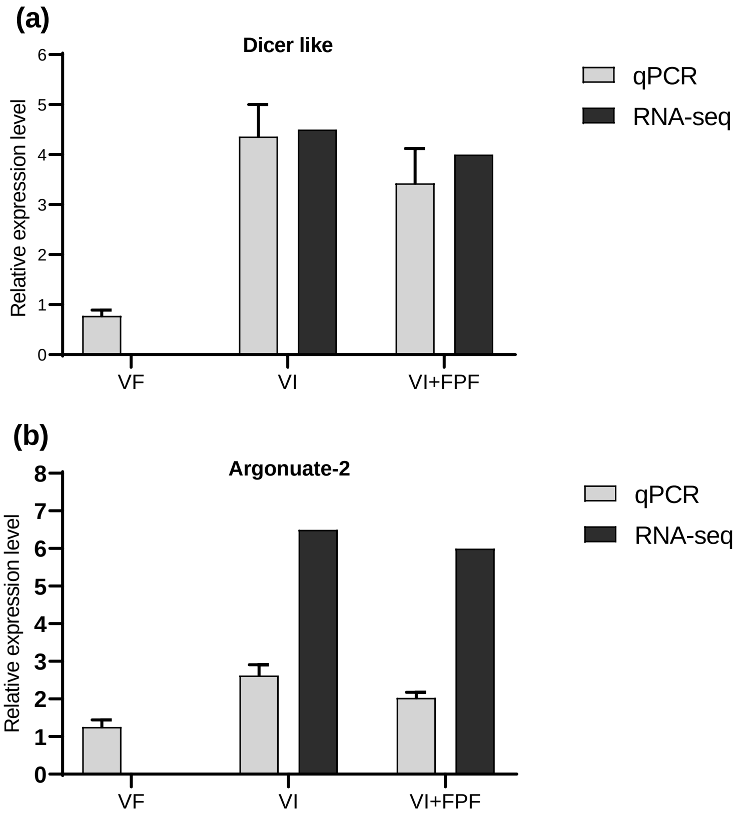

Figure S2. Expression of two genes (a: *Dicer-like*; b: *Argonaute-2*), as quantified by qPCR in previous research followed the same experimental conditions [4,5] and by the RNAseq data in the current study.

References

1. Chen, S.; Zhou, Y.; Chen, Y.; Gu, J. Fastp: An Ultra-Fast All-in-One FASTQ Preprocessor. *Bioinformatics* **2018**, *34* (17), i884–i890. https://doi.org/10.1093/bioinformatics/bty560.
2. Kim, D.; Langmead, B.; Salzberg, S. L. HISAT: A Fast Spliced Aligner with Low Memory Requirements. *Nat. Methods* **2015**, *12* (4), 357–360. https://doi.org/10.1038/nmeth.3317.
3. Bushmanova, E.; Antipov, D.; Lapidus, A.; Prjibelski, A. D. RnaSPAdes: A de Novo Transcriptome Assembler and Its Application to RNA-Seq Data. *Gigascience* **2019**, *8* (9). https://doi.org/10.1093/gigascience/giz100.
4. Al Naggar, Y.; Paxton, R. J. Mode of Transmission Determines the Virulence of Black Queen Cell Virus in Adult Honey Bees, Posing a Future Threat to Bees and Apiculture. *Viruses* **2020**, *12* (5), 535. https://doi.org/10.3390/v12050535.

5. Al Naggar, Y.; Paxton, R. J. The Novel Insecticides Flupyradifurone and Sulfoxaflor Do Not Act Synergistically with Viral Pathogens in Reducing Honey Bee (Apis Mellifera) Survival but Sulfoxaflor Modulates Host Immunocompetence. *Microb. Biotechnol.* **2021**, *14* (1), 227–240. https://doi.org/10.1111/1751-7915.13673.
